# SOURCES OF INTRINSIC MOTIVATION IN LATER ADULTHOOD: A QUALITATIVE ANALYSIS

**DOI:** 10.1093/geroni/igac059.2169

**Published:** 2022-12-20

**Authors:** Melinda Heinz, Nathan Benton, Laura Gleissner

**Affiliations:** Upper Iowa University, Fayette, Iowa, United States; Upper Iowa University, Fayette, Iowa, United States; Upper Iowa University, Fayette, Iowa, United States

## Abstract

A distinct sense of purpose and motivation is increasingly recognized as a contributing factor in successful aging and prolonged health in older adults. While prior studies have established the relationship between intrinsic motivation, defined purpose, and health outcomes, it is essential for care providers and researchers to understand the underlying sources of purpose and motivation for older adults. This study examined qualitative data collected over a two-week period from a sample of older adults (N = 15) in the Midwest. Participants documented daily, through written journal entries and photography, aspects of their lives that they felt illustrated sources of purpose and motivation. The journal entries were then transcribed, aggregated, coded, and analyzed for potential themes. Two researchers coded the data; and a codebook was used to increase coding consistency. Using latent thematic analysis, three distinct themes developed from the dataset. First, engagement in behaviors and activities that incorporated aspects of mindful practices, particularly sensory awareness and grounding, proved to be motivating for many of the participants. The second theme discovered that participants found purpose in active social participation, where a subtheme regarding the impact of technology on maintaining relationships emerged. In the third theme, a goal-oriented mindset proved to be a key aspect of ongoing motivation, with a particular focus on cognitive and physical development, lifelong learning, and planning for the future. Overall, the data pointed to three distinct themes that can be used to foster ongoing opportunities for greater meaning in the lives of older adults.

